# Letter to the Editor regarding the article “Outbreaks of Legionnaires’ Disease and Pontiac Fever 2006–2017”

**DOI:** 10.1007/s40572-021-00319-3

**Published:** 2021-05-18

**Authors:** Paul D. Rockswold, Gabrielle Bernier

**Affiliations:** 1 Cogency Medical in Baltimore, Baltimore, USA; 2 College of Charleston, Charleston, USA

Dear Water and Health Section Editor, T Wade,

We read with interest the article by Hamilton titled “Outbreaks of Legionnaires’ Disease and Pontiac Fever 2006–2017” [1]. Because the Centers for Disease Control and Prevention (CDC) reports a 5.5-fold increase between 2000 and 2017 [2••], we also share the concern about the increasing number of reports of legionella-related disease. However, Hamilton’s article, similar to the 2014 review by Walser [3] covering 2001–2012, presents a biased summary of reality. Based upon a small sample of cases, it reinforces a narrow view that cooling towers are the predominant source of cases of Legionnaires’ disease. It misses the need to address the broader issue.

The manner in which these two articles have been subsequently cited in some publications is quite concerning. Like a classic game of telephone, they drop critical modifying information. In the end, the original meaning has shifted. Using Hamilton [1] as its reference, the GroveWare Technologies [4] report makes two claims: One, that “cooling towers are the most commonly confirmed source of the bacterium that causes Legionnaires’ disease outbreaks,” and, two, that they “are responsible for the majority of outbreak deaths.” These claims are too generalized because the authors leave out the vital modifying phrase, “in the current literature review.” These statements, without the vital modifying phrase, incorrectly reinforce a narrow view.

Llewellyn’s [5] abstract echoes the narrow view, starting with, “Cooling towers (CTs) are a leading source of outbreaks of Legionnaires’ disease…” This idea is the catalyst to perform and report on a nationwide cooling tower sampling study. Showers and whirlpool spas are mentioned only once in the body of the paper. Cassell [6•] has a broader approach, recognizing that “Outbreaks tend to be associated with contaminated cooling towers and potable water sources…” She appropriately expands the position with, “…but the majority of legionellosis cases are not known to be associated with a common exposure,” and “that natural environmental reservoirs may have a greater influence on sporadic legionellosis cases than previously thought.”

Hammami [7] reports a Legionnaires’ disease cluster in Belgium. In the outbreak, the patients had *Legionella pneumophila* serogroup 1. Six cooling towers and, later, a truck wash and car wash were considered as possible sources. The truck wash samples were negative for legionella. The car wash samples were “positive up to 590,000 cfu/l [colony forming units per liter of water] of *L. pneumophila* serogroup 1,” which matched the serogroup found in the patients. The six cooling towers found only serogroups 2 to 14 at much lower concentrations of 500 and 2600 cfu/l. The car wash was the only source with the serogroup that matched the patients. However, the report concluded that a cooling tower with false negative legionella tests was the source of the outbreak [7]. The results did not match the narrow view that cooling towers are responsible for most outbreaks. Additional hypotheses must be considered when this occurs. Could this have resulted from a common water source to the community, reservoirs, storage sites, or the distribution system? Could maintenance on the distribution system have produced multiple simultaneous seeding events? How much did confirmation bias influence Hammami’s conclusion? How long will we maintain this perspective, dismiss evidence that does not align with it, miss the bigger picture, and publish other articles that reinforce the narrow view?

It is inappropriate to generalize the findings of Hamilton’s [1] systematic review to the majority of cases of Legionnaires’ disease. Although their search criteria may be relevant to summarize outbreaks that are both investigated and then published in the literature, the criteria miss the great majority of Legionnaires’ disease. An outbreak consists of two or more temporally and geographically related cases compared to the five cases required by Hamilton. The CDC form for case reports [8] only provides options for use of respiratory equipment, pulmonary conditions, and spa and whirlpool exposure. Cooling towers are not an available option. Likewise, other known sources, such as showers, hot water tanks, grocery store misters, car washes, fountains, sprinkler systems, and even drinking water, cannot be specifically annotated.

The published Legionnaires’ disease outbreaks represent only a small fraction of cases; therefore, publication bias is potentially an important concern. Hamilton reports 80 percent, and the CDC [9, 10] reports 96 percent of cases are sporadic. As such, they are not usually investigated. If only a couple of cases in an outbreak are identified and investigated, they might be summarized by periodic CDC reports, but a public health officer is unlikely to submit that to the peer-reviewed literature. If it were submitted, an editor would be unlikely to publish it since it would seem to offer little to no new medical knowledge. This preferentially leads to more literature that may erroneously reinforce the narrow view. This is even true in a well-done systematic review, which cannot eliminate bias in the content of original publications.

Vastly more cases exist than are reported or included in the literature. Hamilton’s cases are a tiny fraction of the overall number of cases. Hamilton’s cases cover a 22-year period from January 1, 1996 to December 31, 2017, with only 3642 total confirmed cases from Europe, North America, Asia, and New Zealand and Australia [1]. During 2017, the United States by itself had 6221 reported cases [11]. The CDC information page states there were “nearly 10,000 in 2018” [12]. Europe alone had 11,343 reported cases during 2018 [13], more in 1 year than triple the amount that Hamilton’s report covers in 22 years. In their report, GroveWare Technologies claims that “estimates suggest as many as 70,000 people may suffer from Legionnaires’ disease each year in the United States alone” [4]. By comparison, Hamilton includes an average of 166 cases per year. This is only 0.2 percent of that 70,000. It is inaccurate to conclude that this small subset is generally representative of all cases.

Current day multi-state outbreaks of food borne disease show the value of broader views. An epidemic in a city might be traced back to peanut butter or strawberries from a certain store. What might not be apparent with a narrow view is that it might be occurring at the same chain in other cities. When an even bigger picture is considered, multiple states might be identified and that the ultimate source was from a single producer with contaminated product.

Driven by a series of articles based on analyses of limited numbers of outbreak cases, the problem of narrow view may be occurring again, suggesting that cooling towers are the greatest contributors to Legionnaires’ disease. This narrow view does not consider other important sources such as the water supply system and many other commonly accepted sources of exposure. Canright reports that both the New York State (NYS) Department of Health and the New York City (NYC) Council in 2015 passed emergency regulations focused on cooling towers that were fully ratified in 2016. The regulations include registration requirements, posting of information, installation of additional equipment, water sampling and reporting requirements, recurring inspection requirements, and more [2••]. Implementation of the new regulations was costly in terms of human resources, equipment, chemicals, and the additional taxes required to support enforcement of these directives. Claims are now being made that “cooling tower registries are widely considered one of the best practices in preventing and improving the response to legionellosis outbreaks” [4]. In 2017, New Orleans followed the example from New York and created regulations focused primarily on cooling towers [2••]. Despite this huge, continuing cost investment in NYC and NYS, the returns have not materialized. The year-end totals of reported cases in 2016, 2017, and 2018 were 268, 435, and 654 in NYC; in NYS, they were 463, 587, and 770. One element of causality is that a “cause” leads to a specific “effect.” Removing the cause should remove the effect or make it less likely [14]. Fixing cooling towers—the “cause”—did not result in decreasing numbers of cases, but rather the opposite.

In summary, narrow views and actions reinforced by these types of papers result in programs that are implemented at great expense, but without resolving the problem (effect). As with the examples of food borne illness, certain repeated and incomplete messages misdirect our public health and other resources toward solutions that do not correct what proponents claim. It is time to rethink our current positions on Legionnaires’ disease to develop a more holistic response.

## References

[1] Hamilton KA, Prussin AJ, Ahmed W, Haas CN (2018). Outbreaks of Legionnaires’ disease and Pontiac fever 2006-2017. Curr Environ Health Rep.

[2] •• Canright M, Rebullida M, Kahn D, Martinelli J. Legionella regulations in New York: how much progress have we made? The Synergist, the official publication of AIHA. 2019;20:4 **This article illustrates that interventions based on a narrow view may be ineffective despite their cost.**

[3] Walser SM, Gerstner DG, Brenner B, Höller C, Liebl B, Herr CE (2014). Assessing the environmental health relevance of cooling towers—a systematic review of legionellosis outbreaks. Int J Hyg Environ Health.

[4] Groveware Technologies and NSF Health Sciences (2020) Urban Sustainability Directors Network (USDN): Improving public health and sustainability outcomes: electronic registration systems for cooling towers

[5] Llewellyn AC, Lucas CE, Roberts SE, Brown EW, Nayak BS, Raphael BH. Distribution of legionella and bacterial community composition among regionally diverse US cooling towers. PLoS One. 2017;12(12):1–16. 10.1371/journal.pone.0189937. 10.1371/journal.pone.0189937 PMC5738086 29261791

[6] • Cassell K, Gacek P, Warren JL, Raymond PA, Cartter M, Weinberger DM. Association between sporadic legionellosis and river systems in Connecticut. J Infect Dis. 2018;217(2):179–87. 10.1093/infdis/jix531**This article demonstrates had additional information can be obtained when exploring hypotheses outside the narrow commonly quoted sources of exposure. More of this exploration needs to happen.** 10.1093/infdis/jix531 PMC6279145 29211873

[7] Hammami N, Laisnez V, Wybo I, Uvijn D, Broucke C, Van Damme A, Van Zandweghe L, Bultynck W, Temmerman W, Van De Ginste L, Moens T, Robesyn E (2019). A cluster of Legionnaires’ disease in Belgium linked to a cooling tower, August-September 2016: practical approach and challenges. Epidemiol Infect.

[8] Centers for Disease Control and Prevention (2020) National center for immunization and respiratory diseases legionellosis case report, in CDC 52.45 (E), S508 Electronic Version, January

[9] Centers for Disease Control and Prevention (2011). Legionellosis—United States, 2000-2009. MMWR Morb Mortal Wkly Rep.

[10] Garrison LE, Kunz JM, Cooley LA, Moore MR, Lucas C, Schrag S, Sarisky J, Whitney CG (2016). Vital signs: deficiencies in environmental control identified in outbreaks of legionnaires’ disease—North America, 2000-2014. MMWR Morb Mortal Wkly Rep.

[11] Barskey A, Lackraj D, Tripathi PS, Cooley L, Lee S, Smith J, Edens C (2020). Legionnaires’ disease surveillance summary report, United States 2016-2017. MMWR Surveill Summ.

[12] Legionnaires’ disease and Pontiac fever (2018). In: Legionella. Centers for Disease Control and Prevention. https://www.cdc.gov/legionella/about/history.html. Accessed 31 Oct 2020.

[13] Legionnaires’ disease. In: ECDC Annual epidemiological report for 2015. European Centre for Disease Prevention and Control. Stockholm. 2017; https://ecdc.europa.eu/sites/portal/files/documents/AER_for_2015-legionnaires-disease_0.pdf. Accessed 31 Oct 2020.

[14] Woodward M (2005). Epidemiology: study design and data analysis.

